# Insights on mental health when living with rheumatoid arthritis: a descriptive qualitative study of threads on the Reddit website

**DOI:** 10.1186/s41927-020-00163-2

**Published:** 2020-11-26

**Authors:** Jamie Y. E. Park, Alyssa M. Howren, Eileen Davidson, Mary A. De Vera

**Affiliations:** 1 Faculty of Pharmaceutical Sciences, University of British Columbia, 2405 Wesbrook Mall, Vancouver, BC V6T 1Z3 Canada; 2 Collaboration for Outcomes Research and Evaluation, Vancouver, BC Canada; 3 Arthritis Research Canada, Vancouver, BC Canada

**Keywords:** Rheumatoid arthritis, Mental health, Qualitative research, eHealth

## Abstract

**Background:**

Reddit is a highly visited social news and discussion website where individuals anonymously ask questions, post opinions and share experiences, which provide a valuable pool of publicly available data. Our objective was to systematically search and analyze threads on the social news website, Reddit, to understand experiences of individuals with rheumatoid arthritis (RA) regarding their mental health.

**Methods:**

We conducted a patient-oriented descriptive qualitative study. We identified threads from two subreddits, “r/Thritis” and “r/Rheumatoid”, using keywords such as “mood”, “mental health”, “stressed”, “depressed”, “anxious” over a 1-year period between June 2018 and June 2019. For included threads, we extracted the title, original post, and corresponding comments and responses. We applied thematic analysis using an inductive approach.

**Results:**

Of 81 threads identified, we included 27. We identified four themes: 1) *Navigating the management of RA* explores how the physical impacts of the disease, lack of health resources/support and the complexity of medications affect mental health; 2) *Experiencing impact on relationships and social isolation* includes experiencing misconceptions of RA, feeling misunderstood and feeling guilt; 3) *Experiencing loss*, touches on the helplessness brought by challenges with performing self-defining activities such as self-care, work, and childbearing/parenting; and finally, 4) *Experiencing emotional struggles* captures how tension between fighting through and despair has led some to suicide ideation and thoughts of death.

**Conclusions:**

Online forums and communities such as Reddit have created opportunities for individuals with RA to share experiences on mental health matters, which they may not necessarily be able to share with others.

## Background

Rheumatoid arthritis (RA) is the most common autoimmune arthritis, affecting approximately 0.3 to 1.0% of the population globally and is disproportionately diagnosed among females [1]. RA is characterized by inflammation of the joints causing pain and potential joint damage, and inflammation can also affect other organ tissues [2]. Several comorbidities, such as cardiovascular disease, diabetes, and chronic obstructive pulmonary disease are associated with RA [3–7].

In addition to physical manifestations of RA and their impacts on patients, it is also important to consider mental health impacts. Mental health can be defined as the state of one’s mental well-being and involves an individual’s ability and capacity to cope with their environment, process and solve problems, form meaningful relationships, choose their own level of social participation, and lead a personally fulfilling life [8, 9]. Prior research, particularly those using qualitative approaches, have identified aspects of poor mental health (e.g. as a theme or sub-theme) associated with RA [10, 11]. In 2017, Machin et al. [12] explored perceptions of anxiety and depression through qualitative interviews conducted with RA patients recruited from a community clinic in the UK. Most of the 14 participants acknowledged the negative impact of RA on their mental health and a number of participants normalized their depression and anxiety. However, to our knowledge, studies that have directly investigated the broader concept of mental health in individuals with RA are limited. Internet-based communities and online forums where patients discuss mental health experiences, seek advice, and receive peer-to-peer support may represent an authentic source of data for informing such questions on the impacts of mental health in RA. As such, our objective was to conduct a qualitative study utilizing the popular social news website, Reddit, to understand experiences of individuals with rheumatoid arthritis (RA) regarding their mental health.

## Methods

### Patient partnership

This study collaborated with a patient partner from the Arthritis Patient Advisory Board (APAB) of Arthritis Research Canada to ensure that study findings are informed by the patient perspective. The patient partnership role was to collaborate in study decision-making [13], and specifically spanned data analysis, interpretation, manuscript writing, and knowledge dissemination.

### Study design and data source

We conducted a descriptive qualitative study of threads on the social news website, Reddit, as shared within its online community. The use of online data, such as Reddit, where individuals can ask questions, discuss opinions and experiences provides a valuable pool of publicly available data to explore the impact, management and the unmet needs of individuals with RA regarding their mental health. Reddit is a highly visited social news website, where submitted content (“posts”) are organized according to subject in subreddit communities. There are over 430 million average monthly active users, over 130 thousand active communities and 21 billion average screen views per month as of March 2020 [14]. This website has become a popular venue for individuals suffering from mental disorders to engage in written communications [15]. Reddit ensures anonymity and does not have a limited word count, which may contribute to individuals to engage in honest discussions, which may not be applicable in other social media sites such as Facebook [16–19]. Currently, there is a growing amount of literature on utilizing Reddit to explore topics and unmet needs of specific health communities [20, 21].

### Search strategy and data extraction

Our search was guided by our research questions, which included what information and stories are users sharing or asking on the social news website, Reddit. Since there are no specific subreddit threads on the topic of RA and mental health, we searched for eligible threads on two subreddits, “r/Thritis” and “r/Rheumatoid,” over a 1-year period June 21st, 2018 and June 21st, 2019. First, threads were identified using keywords including “mood”, “mental health”, “stressed”, “depressed”, “anxious”. Second, posts were reviewed and included in the analysis if: 1) the thread consisted of an original post and at least one comment/response; 2) the original poster indicated having RA and sharing a lived experience, concern, or question about mental health. For each included post, we extracted the following information: title, word counts for original post and comments, number of comments, and number of unique users.

### Analysis

We conducted a descriptive qualitative study to interpret the mental health experiences and perspectives of individuals living with RA. We specifically applied a thematic analysis, which involved initial line-by-line or segment-by-segment coding followed by the construction of categories, and then the development of themes [22–24]. When constructing categories and themes, we applied an inductive approach by deriving results directly from the data as opposed to employing an existing theory or framework. We constructed categories and themes from data through the identification of repetition and patterns observed within the dataset [23, 25]. Our analysis also used the constant comparative method, an iterative process drawn from the principles of Grounded Theory that makes comparisons between initial codes, categories, and themes [26]. One study author (JP) conduced the initial line-by-line coding. Afterwards, all study authors (JP,AH,ED,MDV) were actively involved in the formation of categories and the themes and collaborated to reach a consensus for the final reporting. Study authors also constructed a thematic framework to illustrate the relationships among categories and themes when understanding mental health impacts among individuals living with RA. We used NVivo 12 (QSR International) to support all analyses.

### Ethics

According to the University of British Columbia’s Behavioural Research Ethics Board, this research did not require an ethics approval due to the use of publicly available information from a social network site (i.e., one does not need a social network account or password to access) and the absence of other non-public source to obtain the data.

## Results

In total, 81 threads including 33 threads from r/Thritis, 48 from r/Rheumatoid were identified. After applying the inclusion criteria, 27 threads including 6 from r/Thritis and 21 from r/Rheumatoid, were included in the analysis (Table 1). In total, there were 251 unique users that participated in the threads, including 51 from r/Thritis and 200 from r/Rheumatoid. We noted an average of 9.5 unique users providing comments and/or replies to the original post. We also determined that the average word count across original posts and comments/replies was 357.

**Table 1:** Summary of subreddit threads downloaded and included

	r/Thritis	r/Rheumatoid
Subscribers	4200	6000
Threads downloaded^a^	33	48
Threads included^a^	6	21
Total numbers of unique commenters	51	200
Average number of total comments	14	18
Average number of total users that commented^b^	9	10
Word count of post (excluding title)	407	306

^a^ Between June 21st 2018, and June 21st, 2019

^b^Exclude autobots (e.g. converter-bot)

From our thematic analysis, we identified four themes – 1) Navigating the management of RA; 2) Experiencing impact on relationships and social isolation; 3) Experiencing loss; and 4) Experiencing emotional struggles. These are described as follows. In addition, a summary of the themes, categories and additional representative quotes are shown in Table 2.

**Table 2:** Summary of the themes, categories, representative quotes

Themes	Categories	Representative Quotes
**1. Navigating the management of RA**	1. How symptoms of RA impact mental health	*Fatigue* - “What’s hitting me the hardest is the fatigue, I have no energy to even get out of bed.” (**rRheumatoid**)
		*Pain* - “Can I hope to have pain free hands ever again?” (**rThritis**)
	2. Lack of health resources and support	*Finding resources and support* - “I really would like to join a local RA support group but cannot find any here. I am old school, I like talking to people!” (**rRheumatoid**) *Struggling to find doctors who understands* - “At first she was great but the past year there is just a severe lack of communication and I feel she really isn’t listening to me. My husband who just recently started going to visits with me feels the same.” (**rThritis**)
	3. Complexity of medications	- “Sometimes I have a brief cry just thinking about sitting in my room, alone, injecting MTX into my fatty tissue. It’s not the pain or the needle... Just the reality of it, and the fear of side effects, and just going through this all alone.” (**rRheumatoid**) - “I’ve been so discouraged lately about my arthritis and all the troubles and stresses it brings me that I stopped taking my meds.” (**rRheumatoid**)
**2. Experiencing impact on relationships and social isolation**	1. Experiencing misconceptions of having RA	- “Just recalling all the pressures and hate I got at your age from people calling me lazy, or accusing me of poor sleeping habits, lack of exercise, or whatever other homespun shit they could think of infuriates me.” (**rThritis**) - “I’ve had people tell me to “drink this tea” and that my meds are bad for me, but none of them have had RA. The worst thing I’ve experienced was a kid telling me I couldn’t have RA cuz he does but doesn’t need medication.” (**rRheumatoid**)
	2. Feeling not understood	*Family not understanding* - “My parents are pushing me super hard to get a job and do extracurricular activities but I can’t even get through school most days without having to leave or go to bed as soon as I get home, leaving me without a social life.” (**rThritis**) *Co-workers not understanding* - “They know about my RA and they know about my MTX injections. However, they haven’t been that supportive. There’s a lot of blank stares when I share anything about my RA.” (**rRheumatoid**)
	3. Feeling guilt	- “I don’t want my parents to be wasting their money on me and my medical problems (I also have a heart condition) that’s the part that makes me feel so damn guilty” (**rRheumatoid**) - “Now I feel guilty because I can’t do anything at the moment and my darling kids and husband are picking up the slack for me.” (**rThritis**)
**3. Experiencing loss**	1. Daily functioning and self-care	- “You can’t DO things that most people in their 20’s want to do, which is enjoy life, and getting that higher education your folks want you to have, going camping, or on trips, a lot of this can feel very out of reach.” (**rRheumatoid**) - “Going to the gym is so relaxing and reliving and just takes all the nonsense away but I can barely walk.” (**rThritis**)
	2. Work disability	- Breaks my heart to know I’m not going to be able to do the sorts of things I desperately want to do with my career, but I had to make choices based on where I could do the most good while still being able to live my life. (**rRheumatoid**) - I teach and finals are next week, my hands hurt so bad, no idea how I am going to type them, much less grade them. Supposed to prep all weekend but all I can seem to do is stare off into space, try not to throw up and wonder all the ways my life is going to change. (**rRheumatoid**)
	3. Family planning and parenting	- I don’t want to show my family, my kids/ hubby are really supportive but I don’t want to freak them out so I am showing them that I GOT THIS but inside I am frightened and trying to accept and adjust. (**rRheumatoid**) - I developed RA after pregnancy so it’s really hard to deal with the disease, taking care of a child, and also weighing whether or not I should have any more children. (**rRhematoid**)
**4. Experiencing emotional struggles**	1. Fighting through	- “Sometimes you just have to push through (with the agreement from your doctors). This takes a lot of physical and mental energy, and you’re likely to be wiped out afterwards, but it is an effective coping mechanism for some people.” (**rThritis**) - “I have a system to help myself get over that bad feeling when I wake up in pain. And I know a lot of people have a system, but some people don’t. I want to share mine for those who haven’t found a way to help themselves turn from “this is so depressing, why must I always hurt, today is the worst” mentality to one of “I got this, so what if I’m in pain!? Today is going to be good because I’ll make it good!” (**rRheumatoid**)
	2. Having suicidal thoughts and patterns	- “So much of my life is decided for me by my physical inability, and I refuse to live in a state in which I am unable to care for myself. Death is not the worst part of life, nor even the worst thing that can happen to me, and I’m not scared of it at all. I’ve decided to die about the time I no longer have the physical agency to make that decision for myself.” (**rThritis**) - “I’ve spent quite a few evenings over the past month in my room. Suffering from an anxiety attack and at times, suicidal thoughts.” (**rRheumatoid**)

### Theme 1: navigating the management of RA

The first theme captures how living with RA including dealing with, and managing the disease particularly affects users’ mental health and constitutes three categories: 1) how symptoms of RA impact mental health; 2) lack of health resources and support; and 3) complexity of medications. For example, fatigue was significant and limits the ability to “*keep up with the most minimal demands of society*” (**rThritis**). One user shared that the “*depressing part about [fatigue] is realizing how useless [they are].”* (**rThritis**). Although users shared efforts to overcome fatigue, they also share that “*any sense of normalcy would last six weeks at most and cost me thrice the time in sleep”* (**rThritis**). Similarly, with pain, users mentioned that they are “*in so much pain and just not sure what to do.*” (**rThritis**). Users also discussed struggles with managing RA in terms of challenges with finding resources and “*finding [a health care professional] who understands [RA patients]*” (**rThritis**). Many users also expressed difficulties with finding age-specific resources, with one noting that when they “*read the health magazine it’s all older people who have lived their life and now they have RA*” (**rThritis**). Users expressed their dissatisfactions with their doctors when they “*scratch [ … ] their head about what to do*” (**rThritis**) or are experiencing a “*lack of communication*” (**rThritis**). Finding a new doctor can also have a toll on their mental health since “*explaining everything for each new person can get tiring*” (**rRheumatoid**). Another aspect of living with RA that had an impact on mental health was managing the disease, particularly with medications, in terms of finding optimal therapy, feeling overwhelmed with number of medications, and experiencing fear of side effects. Users mentioned that “*it is defeating to have to try so many med[ications] and not have found one that works*” (**rThrtitis**). Taking medications was also discussed with one user sharing that they felt “*discouraged lately about [their] arthritis and all the troubles and stresses it brings [them]*” (**rRheumatoid**). As well, some users who had read information online shared feelings of concern, particularly with initiating these medications as captured by this quote: “*[the medications] sound[ed] scary and [they were] worried that the side effects will be worse than the disease*” (**rRheumatoid**).

### Theme 2: experiencing impact on relationships and social isolation

The second theme focuses on how RA affects mental health through others’ preconceptions and the related impact on relationships. Three categories comprised this theme: 1) experiencing misconceptions of having RA; 2) feeling misunderstood; and 3) feeling guilt. Misconceptions directed at individuals living with RA were perceived by users as negative judgement and unrealistic advice. For example, one user expressed being told “*most people have some arthritis, so I should learn to deal w/it.”* (**rThritis**) while another shared that they felt being seen as “*lazy*” and “*over exaggerating the pain and exhaustion*” (**rRheumatoid**). Users also shared receiving irrelevant, sometimes harmful advice including being told that “*no DMARDs, biologics, or steroids have a place in the treatment of RA*” (**rThritis**). Perceived misconceptions were closely related to users feeling misunderstood by other people, including family members and co-workers, who may not comprehend the nature of a chronic disease like RA. One user described the challenge of others not understanding the difference between acute and chronic diseases as “*healthy people see the “win condition” as killing the disease. My win condition is maintaining the status quo for as long as possible*” (**rRheumatoid**). A lack of understanding regarding RA can stress relationships and create a sense of loneliness or disconnect, especially when family and friends “*cannot understand why [they] are so tired all the time and why [they] cannot just “push through” the pain*” (**rThritis**). Lastly, the third category describes the feelings of guilt held by users with RA, often not wanting to be a burden, and “*feel[ing] guilty because [they] cannot do anything at the moment and [their loved ones] are picking up the slack for [them]*” (**rThritis**). Feelings of guilt also arose from family spending resources for RA care as shared by this user: “*I don’t want my parents to be wasting their money on me and my medical problems [ … ] that’s the part that makes me feel so damn guilty*” (**rRheumatoid**).

### Theme 3: experiencing loss

The third theme captures how individuals with RA felt a sense of loss, which can be ongoing process given the chronic nature and unpredictability of RA. This theme is characterized by users with RA as feeling “*stuck in this confusing place where I’m not sure what to do*” (**rRheumatoid**) as well as feeling “*hopeless and lost most days*” (**rThritis**). The experience of loss touched on helplessness brought by the inability or challenges with performing self-defining activities including self-care, work, and childbearing or parenting. Due to the symptoms of RA and functional limitations, many users expressed having to manage their expectations and shared how RA impacted activities of daily living, from exercise to “*having trouble holding [a] coffee cup without pain or carrying grocery bags*” (**rRheumatoid**). With regard to work, individuals with RA described being scared and worried about their future. Indeed, this uncertainty affected the mental health of some users, with one sharing “*the more I think about how bad it is, how worse it will get, how it’ll ruin my career, and how many joints I’ll inevitably need to get replaced, the more depressed I get*” (**rRheumatoid**). RA may also lead to individuals deciding to make sacrifices in their careers in order to prioritize their health. Lastly, the third category describes how the sense of loss associated with RA also extended to childbearing/family planning or parenting. Users who had children shared challenges with parenting sharing that it is “*really hard to deal with the disease, tak[e] care of a child, and also [weigh] whether or not I should have any more children*” (**rRheumatoid**). Not only does the management of RA detract from the ability to care for their current children, but it also creates hesitancy and worry around future pregnancies, with women expressing “*wanting to have more kids but being scared of how I’ll handle it*” (**rRheumatoid**).

### Theme 4: experiencing emotional struggles

The fourth theme describes the emotional burden experienced by individuals living with RA, particularly the ongoing tension between pushing through and feelings of despair, and in some cases, suicidal ideation. Two categories build the fourth theme: 1) fighting through; and 2) having suicidal thoughts and patterns. The concept of fighting through captures users’ accounts of wanting to push through both the physical and mental impacts of RA. To get through the day or situation, users with RA reflected on needing to put on a façade, where the “*thought of going in and smiling and pretending everything is fantastic is so exhausting*” (**rRheumatoid**). This seems to be a reoccurring notion that resonates with many diagnosed with RA as others also described mechanisms to cope with and overcome this challenge. However, for some individuals, the emotional pain evolved into reflections on mortality and suicidal ideation, as captured by the second category. Indeed, this aspect emphasizes significant suffering that can lead to suicidal thoughts and patterns, with one user voicing “*It’s been so bad recently I’ve had serious suicidal thoughts, because it feels like it will all be easier*” (**rRheumatoid**).

### Framework for understanding RA and mental health

**Fig. 1:**
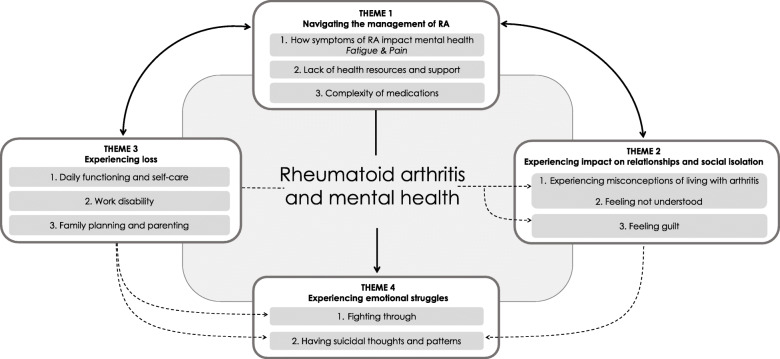
Conceptual framework to illustrate the connections among themes and categories that collectively describe how RA impacts mental health. Solid arrows (either bidirectional or unidirectional) indicate relationships between themes. Dashed arrows show plausible connections among themes and categories. Categories listed in the same shaded box within a theme are closely related

A thematic map was created to understand the relationships amongst the themes and categories (Fig.1: Conceptual framework to illustrate the connections among themes and categories). The framework illustrates the reciprocal relationship that theme 1 (navigating the management of RA) has with both theme 2 (experiencing impact on relationships and social isolation) and theme 3 (experiencing loss). This relationship is evidenced by, for example, users seeking help from healthcare providers (theme 1) and relaying a “*severe lack of communication and [feeling] she really isn’t listening to me*” (**rThritis**), which connects to feeling not understood (theme 2). In addition, the effects of RA (theme 1) lead to a sense of loss (theme 3) through marked impacts on daily functioning, work, and family, and overall “*finding out that you can’t do the same things as you used [to]*” (**rRheumatoid**). These losses may initiate looking into additional health care supports (e.g., mental health, occupational), or in some specific cases, reflecting on the effect of medications: “*My option to have children is pretty much thrown out the window because of all the meds I’m on*” (**rThritis**). The unidirectional relationship between theme 1 and theme 4 (experiencing emotional struggles) is highlighted by a need to fight through pain, fatigue and management of RA, with one individual sharing “*weekdays I’m forced to get 6.5 hours of sleep and it feels like I have the flu and every movement aches and just feels heavy”* (**rThritis**). In more severe cases, having RA may gradually lead to having suicidal thoughts (theme 4), with one user stating that they “*could literally fill a book about every way arthritis makes [them] depressed and borderline suicidal at times*” (**rRheumatoid**). Finally, this framework also depicts potential connections among categories from themes 2, 3, and 4. For instance, there is perhaps a sense of guilt (theme 2) arising from a loss of abilities such as parenting (theme 3), with one parent noting “*today I barely saw my kids even though I was off work because I fell asleep on the couch due to the fatigue*” (**rRheumatoid**).

## Discussion

The social news website Reddit, where individuals can engage with each other, share experiences, and exchange information, provides a potentially rich source of data for research to understand experiences of specific patient groups. This patient-oriented qualitative study applied a descriptive thematic approach to analyze threads from Reddit with the aim of understanding the experiences of individuals with RA regarding their mental health. Four themes were constructed from the analysis and were characterized as ‘navigating the management of RA’, ‘experiencing impact on relationships and social isolation’, ‘experiencing loss’, and ‘experiencing emotional struggles.’ These findings were interpreted further to develop a thematic framework that explains relationships among the themes and categories in an effort to enhance our understanding of how mental health-related problems arise and present in individuals with RA. Findings of our study emphasize the close relationship between aspects of RA, specifically symptoms (e.g., pain and fatigue) and disease management (e.g., medications and health resources), and the downstream negative effects on mental health. Indeed, the symptoms of RA and the fact it presents as an invisible illness can lead to a range of continuous losses (e.g., work disability, worries over family planning) and strain relationships, especially when there is a perceived lack of understanding from others as well as personal guilt, which all have substantial implications for one’s mental health. By garnering attention to these phenomena, healthcare providers, families, and communities can recognize and identify opportunities to improve the mental health of patients with RA. Given that a patient’s mental health needs may change from their diagnosis and throughout the course of RA, our findings shed light on the importance of establishing practical recommendations to guide healthcare within rheumatology. Indeed, recognition of the importance of mental health is growing. For instance, in 2018, Leon et al. used a nominal group approach to develop 13 recommendations about psychological needs in RA, which considered key aspects of identification of psychological problems, guidelines for managing psychological problems, approaches to communication with patients, and criteria for referral to mental health professionals [27].

Our particular study was motivated by growing recognition of the importance of psychological impacts of RA, in addition to its physical impacts. Arguably more studied is associated mental health disorders including depression and anxiety, which systematic reviews have shown to be a substantial comorbidity in RA with reported prevalence estimates as high as 39% [28] and 45% [29], respectively. We were particularly interested in the concept of mental health, which encompasses an individual’s overall mental well-being and their level of cognitive-emotional functioning and adaptability, as opposed to the presence or absence of a specific mental illness such as depression [8, 9]. Qualitative studies have not explicitly explored mental health in individuals with RA, nonetheless, some of these have described phenomena similar to those identified in our study. In particular, Toye et al. [30] in 2019 conducted a meta-ethnography of nine qualitative syntheses in RA to characterize experiences of living with RA, with one of the themes, “RA is in control of my body”, relaying the pain, fatigue, unpredictability of symptoms, and the invisibility of this disease. In addition, our theme of experiencing loss is paralleled in the aforementioned meta-ethnography as well as a phenomenological study on lived experiences with RA in the United States by Iaquinta et al. [31] in 2004 that perceptively characterized a process of “grieving while growing.” The impact of RA on emotional wellbeing is also seen in published studies, wherein qualitative findings on the experiences of living with RA depicted themes and categories that capture negative emotions such as fear, guilt, anxiety, and depression [30, 31]. Moreover, in 2017, Poh et al. [10] conducted a descriptive qualitative study in Singapore on experiences of living with RA and noted a theme on “psychological and emotional challenges”, with one participant sharing their thoughts of suicide. Suicidal thoughts and patterns were also discussed in the Reddit forums analyzed in our study and perhaps more so given the anonymity of the platform. Given the data that support our findings, showing the mental health impact of RA and the potential for mental illness and suicidal ideation, it is critical to continue understanding patient’s mental health needs and move further towards better integration of mental health care in the clinical setting.

An important contribution of our paper is a conceptual framework for understanding how RA impacts someone’s mental health, highlighting the interactions between physical, social, and emotional experiences. The connection we have observed between RA symptoms and disease management with aspects of wellbeing is supported by one qualitative study published in 2017 by Machin et al. [12] on perspectives and management approaches for anxiety and depression in individuals in with RA in the UK, as participants shared the negative impact functional limitations and pain had on their mood. Interestingly, in the same study some participants felt that a depressed mood also impacted their disease activity in terms of flares [12]. Although the study by Machin et al. [12] focuses on depression and anxiety, it diverges from our study to primarily discuss barriers and preferences for mental health support. An advantage of our paper are insights into several factors, such family planning, feelings of not being understood and guilt, that provide opportunities for health care providers to support the mental health of their patients with RA. Further, the preliminary framework can be strengthened through incorporating findings from other studies along with future qualitative exploration into the mental health experiences of individuals living with RA.

Strengths and limitations of our study warrant discussion. We demonstrated a systematic approach to identifying and applying a thematic approach to analyzing eligible subreddit threads to understand experiences of individuals with RA regarding their mental health. Currently, there is growing literature utilizing Reddit to explore topics and issues experienced by patients with arthritides [32, 33]. Features of this widely popular and highly accessed platform including anonymity for users, no word limits on entries, as well as engaged subreddit communities make it particularly suited for discussion of potentially sensitive and stigmatizing topics such as mental health. Our approach allowed us to take advantage of these features by drawing from this world-wide, immense pool of data. However, due to the anonymity of the Reddit posts, we were not able to gather demographic information about users. Furthermore, there is also potential selection bias with respect to individuals who may be more likely to use Reddit, which aside from those who have Internet access are those from the US, UK, and Canada and between 18 to 49 years of age, based on recent data on characteristics of Reddit users [34]. As we were also not able to gather information on disease, we cannot be certain of users’ diagnoses or that they truly have RA. However, we systematically searched relevant subreddits (e.g. r/Thritis, r/Rheumatoid) for individuals with RA. Furthermore, given the specificity of an RA diagnosis, we anticipate a high likelihood that original posts are written by individuals experiencing the impacts of the disease. Finally, due to the individual variabilities in expressing one’s mental health, the keywords used to identify eligible posts for this study may not have captured the entirety of eligible Reddit threads.

## Conclusions

Altogether, our study contributes towards a better understanding of interrelations between lived experiences of RA and mental health implications. As with many patients in today’s digital era, individuals with RA look to online communities, such as Reddit, to ask questions and seek information about aspects of RA including disease management and effects on self as well as relationships. Our study not only revealed how these challenges associated with RA impact mental health, highlighting important support roles for healthcare providers, families, and communities, but also identified opportunities to improve the mental health of patients with RA. In particular, our findings highlight the interconnectedness of RA symptoms/disease management and mental health. As well, we provide insights into areas of future research, including family planning and feelings of not being understood and guilt.

## Supplementary information

**Additional file 1.**

## Data Availability

The effect of data sharing is not applicable to this article as no datasets were generated or analysed during the current study.

## Abbreviations

RA: Rheumatoid arthritis
APAB: Arthritis Patient Advisory Board
