# Supplementary material for: Insights on mental health when living with rheumatoid arthritis: a descriptive qualitative study of threads on the Reddit website
Source: BMC Rheumatol. 2020 Nov 26;4:62. doi: 10.1186/s41927-020-00163-2 (PMC7690206; doi:10.1186/s41927-020-00163-2)
Supplement: Supplementary file 1 — Additional file 1. [file 41927_2020_163_MOESM1_ESM.docx]

| Name | Memo Link | Files | References | Created On | Created By | Modified On | Modified By |
| --- | --- | --- | --- | --- | --- | --- | --- |
| Demographics |  | 17 | 47 | Jun 27, 2019 at 4:06:10 PM | JP | Jul 7, 2019 at 12:31:22 AM | JP |
| Lack of needs met |  | 0 | 0 | Jul 1, 2019 at 10:49:59 PM | JP | Jul 1, 2019 at 10:50:14 PM | JP |
| Hard to find help |  | 1 | 1 | Jul 1, 2019 at 10:50:29 PM | JP | Jul 8, 2019 at 3:20:06 AM | JP |
| Hard to find resources relevant to them |  | 2 | 2 | Jul 2, 2019 at 2:24:31 PM | JP | Jul 3, 2019 at 9:37:49 AM | JP |
| No Inperson support group available |  | 1 | 1 | Jul 2, 2019 at 4:37:37 PM | JP | Jul 8, 2019 at 3:21:11 AM | JP |
| New to sharing about their disease |  | 1 | 2 | Jul 2, 2019 at 2:03:18 PM | JP | Jul 8, 2019 at 3:31:04 AM | JP |
| Being apologetic for the readers |  | 2 | 2 | Jul 2, 2019 at 2:05:09 PM | JP | Jul 8, 2019 at 3:22:31 AM | JP |
| First time posting on Reddit |  | 4 | 5 | Jul 1, 2019 at 10:29:06 PM | JP | Jul 8, 2019 at 3:22:28 AM | JP |
| People don't understand |  | 11 | 20 | Jun 27, 2019 at 3:56:15 PM | JP | Jul 7, 2019 at 12:25:59 AM | JP |
| Pregnancy struggles |  | 1 | 1 | Feb 4, 2020 at 7:29:29 PM | JP | Feb 4, 2020 at 7:29:29 PM | JP |
| Provides empathy |  | 8 | 18 | Jul 1, 2019 at 10:45:38 PM | JP | Jul 8, 2019 at 3:54:56 AM | JP |
| Provides empathy - Family Relationship |  | 2 | 3 | Jul 1, 2019 at 10:24:47 PM | JP | Jul 5, 2019 at 3:46:58 PM | JP |
| Provides empathy- understands their concern or trouble |  | 16 | 68 | Jul 1, 2019 at 10:44:13 PM | JP | Jul 8, 2019 at 4:00:55 AM | JP |
| Reporting similar experiences -Different circumstances or disease |  | 12 | 22 | Jun 27, 2019 at 3:49:42 PM | JP | Jul 6, 2019 at 11:45:20 PM | JP |
| Seeking Advice |  | 0 | 0 | Jul 1, 2019 at 10:41:15 PM | JP | Jul 8, 2019 at 3:13:09 AM | JP |
| Asks for advice for treatments |  | 3 | 8 | Jul 1, 2019 at 10:40:59 PM | JP | Jul 7, 2019 at 12:25:23 AM | JP |
| Asks for advice or reassurance |  | 13 | 16 | Jul 1, 2019 at 10:40:36 PM | JP | Jul 8, 2019 at 3:58:46 AM | JP |
| Asks for doctor advice |  | 1 | 1 | Jul 1, 2019 at 10:41:07 PM | JP | Jul 1, 2019 at 10:41:47 PM | JP |
| Strategies and tips to help for sucidial thoughts |  | 0 | 0 | Jul 1, 2019 at 11:26:56 PM | JP | Jul 8, 2019 at 4:09:42 AM | JP |
| Empathy |  | 2 | 3 | Jul 1, 2019 at 11:48:13 PM | JP | Jul 8, 2019 at 4:00:39 AM | JP |
| First year of diagnosis is the scariest part and you need time to get used to it |  | 1 | 1 | Jul 1, 2019 at 11:42:30 PM | JP | Jul 1, 2019 at 11:43:36 PM | JP |
| Meds will start help with time, less pain and function properly |  | 1 | 2 | Jul 1, 2019 at 11:43:41 PM | JP | Jul 2, 2019 at 12:12:36 AM | JP |
| Meds are improving |  | 3 | 6 | Jul 1, 2019 at 11:35:49 PM | JP | Jul 8, 2019 at 4:05:31 AM | JP |
| Online information are not set on stone |  | 1 | 1 | Jul 1, 2019 at 11:30:45 PM | JP | Jul 1, 2019 at 11:35:42 PM | JP |
| Positive thoughts |  | 1 | 11 | Jul 1, 2019 at 11:36:39 PM | JP | Jul 8, 2019 at 4:09:09 AM | JP |
| If you don't like your circumstance, change it |  | 1 | 1 | Jul 1, 2019 at 11:36:50 PM | JP | Jul 1, 2019 at 11:37:03 PM | JP |
| Live your life to the fullest |  | 2 | 3 | Jul 1, 2019 at 11:36:19 PM | JP | Jul 3, 2019 at 10:17:14 AM | JP |
| New and current therapies that can help with your disease |  | 1 | 1 | Jul 1, 2019 at 11:45:15 PM | JP | Jul 1, 2019 at 11:45:44 PM | JP |
| Provides different perspective |  | 1 | 3 | Jul 1, 2019 at 11:28:36 PM | JP | Jul 1, 2019 at 11:39:50 PM | JP |
| Seek HCP help and don't feel ashamed |  | 1 | 3 | Jul 1, 2019 at 11:29:41 PM | JP | Jul 1, 2019 at 11:36:16 PM | JP |
| Stress can make it worse |  | 1 | 1 | Jul 2, 2019 at 12:14:34 AM | JP | Jul 2, 2019 at 12:13:07 AM | JP |
| Will burden family more if disease gets worse bc you didn't take care of yourself |  | 1 | 2 | Jul 3, 2019 at 10:15:17 AM | JP | Jul 3, 2019 at 10:15:10 AM | JP |
| Would help or talk to those who are having suicidial thoughts |  | 1 | 1 | Jul 2, 2019 at 12:25:56 AM | JP | Jul 2, 2019 at 12:26:27 AM | JP |
| Strategies for 'bad days' |  | 0 | 0 | Jul 1, 2019 at 10:51:44 PM | JP | Jul 1, 2019 at 10:51:52 PM | JP |
| Brace |  | 1 | 1 | Jul 1, 2019 at 10:52:36 PM | JP | Jul 1, 2019 at 10:52:43 PM | JP |
| Diclofenac gel |  | 1 | 1 | Jul 1, 2019 at 10:54:22 PM | JP | Jul 1, 2019 at 10:54:34 PM | JP |
| Extra mobility work (ex. yoga tune-up balls) |  | 2 | 2 | Jul 1, 2019 at 10:52:50 PM | JP | Jul 5, 2019 at 9:58:24 PM | JP |
| Heat |  | 2 | 2 | Jul 1, 2019 at 10:55:09 PM | JP | Jul 6, 2019 at 11:20:30 PM | JP |
| Hyaluronic acid injections |  | 1 | 1 | Jul 1, 2019 at 10:54:49 PM | JP | Jul 1, 2019 at 10:55:01 PM | JP |
| Massage |  | 1 | 1 | Jul 1, 2019 at 10:52:10 PM | JP | Jul 1, 2019 at 10:52:20 PM | JP |
| Shower |  | 1 | 1 | Jul 1, 2019 at 10:51:54 PM | JP | Jul 1, 2019 at 10:52:07 PM | JP |
| Strategies for disease management |  | 0 | 0 | Jul 2, 2019 at 5:18:47 PM | JP | Jul 8, 2019 at 3:52:07 AM | JP |
| Acupuncture |  | 1 | 4 | Jul 6, 2019 at 11:52:01 PM | JP | Jul 6, 2019 at 11:54:03 PM | JP |
| Lose weight |  | 1 | 2 | Jul 2, 2019 at 5:19:47 PM | JP | Jul 2, 2019 at 5:20:27 PM | JP |
| Push through the day |  | 1 | 2 | Jun 27, 2019 at 3:53:11 PM | JP | Jun 27, 2019 at 3:55:50 PM | JP |
| Push through can take alot of energy but may be effective |  | 1 | 1 | Jun 27, 2019 at 3:53:27 PM | JP | Jun 27, 2019 at 3:53:27 PM | JP |
| Resources to help with functional limitation |  | 1 | 3 | Jul 7, 2019 at 12:15:47 AM | JP | Jul 7, 2019 at 12:27:23 AM | JP |
| Strategies for fatigue |  | 0 | 0 | Jul 2, 2019 at 12:34:56 AM | JP | Jul 2, 2019 at 12:35:30 AM | JP |
| Asks and advises about mental wellbeing |  | 1 | 2 | Jul 2, 2019 at 12:38:42 AM | JP | Jul 2, 2019 at 12:40:37 AM | JP |
| Diet |  | 2 | 2 | Jul 2, 2019 at 12:36:01 AM | JP | Jul 2, 2019 at 2:57:33 PM | JP |
| Downplaying fatigue |  | 1 | 1 | Jul 2, 2019 at 1:43:17 PM | JP | Jul 2, 2019 at 1:43:35 PM | JP |
| Eliminate as many stressors |  | 1 | 1 | Jul 2, 2019 at 1:58:58 PM | JP | Jul 2, 2019 at 1:59:12 PM | JP |
| Get approved for permanent disability |  | 1 | 1 | Jul 2, 2019 at 1:49:51 PM | JP | Jul 2, 2019 at 1:50:04 PM | JP |
| Have to plan out activities beforehand |  | 1 | 1 | Jul 2, 2019 at 1:47:04 PM | JP | Jul 2, 2019 at 1:47:18 PM | JP |
| Massages |  | 1 | 1 | Jul 2, 2019 at 12:35:30 AM | JP | Jul 1, 2019 at 10:48:59 PM | JP |
| Suggests getting iron checked |  | 1 | 1 | Jul 2, 2019 at 12:40:58 AM | JP | Jul 2, 2019 at 12:41:20 AM | JP |
| Take control of your own treatment |  | 1 | 2 | Jul 2, 2019 at 1:56:37 PM | JP | Jul 2, 2019 at 1:58:32 PM | JP |
| Strategies for flareups |  | 0 | 0 | Jul 1, 2019 at 11:12:18 PM | JP | Jul 1, 2019 at 11:12:22 PM | JP |
| Accupuncture |  | 1 | 1 | Jul 1, 2019 at 11:15:06 PM | JP | Jul 1, 2019 at 11:15:28 PM | JP |
| Alter diet |  | 2 | 2 | Jul 1, 2019 at 11:12:36 PM | JP | Jul 2, 2019 at 2:57:31 PM | JP |
| Ice or heat pads |  | 1 | 1 | Jul 1, 2019 at 11:14:31 PM | JP | Jul 1, 2019 at 11:14:46 PM | JP |
| Medication recommendations |  | 1 | 3 | Jul 1, 2019 at 11:14:07 PM | JP | Jul 1, 2019 at 11:16:22 PM | JP |
| Surgery |  | 1 | 1 | Jul 1, 2019 at 11:16:53 PM | JP | Jul 1, 2019 at 11:17:09 PM | JP |
| taichi |  | 1 | 1 | Jul 1, 2019 at 11:14:53 PM | JP | Jul 1, 2019 at 11:15:15 PM | JP |
| Water activities |  | 1 | 1 | Jul 1, 2019 at 11:14:58 PM | JP | Jul 1, 2019 at 11:15:24 PM | JP |
| Strategies for pain |  | 0 | 0 | Jul 1, 2019 at 10:47:10 PM | JP | Jul 1, 2019 at 10:47:14 PM | JP |
| Biologic monotherapy |  | 1 | 1 | Jul 2, 2019 at 12:05:56 AM | JP | Jul 2, 2019 at 12:06:07 AM | JP |
| Compression glove |  | 2 | 3 | Jul 5, 2019 at 2:33:02 PM | JP | Jul 7, 2019 at 12:24:46 AM | JP |
| diet |  | 4 | 9 | Jul 2, 2019 at 3:00:06 PM | JP | Jul 5, 2019 at 9:47:45 PM | JP |
| Exercise |  | 2 | 2 | Jul 1, 2019 at 10:48:38 PM | JP | Jul 5, 2019 at 8:44:21 PM | JP |
| Get approved for permanent disability |  | 1 | 1 | Jul 2, 2019 at 1:49:27 PM | JP | Jul 2, 2019 at 1:50:07 PM | JP |
| Heat or ice |  | 3 | 5 | Jul 5, 2019 at 2:28:52 PM | JP | Jul 6, 2019 at 11:20:26 PM | JP |
| Jacuzzi |  | 1 | 1 | Jul 2, 2019 at 2:10:26 PM | JP | Jul 2, 2019 at 2:10:38 PM | JP |
| Massages |  | 1 | 1 | Jul 1, 2019 at 10:48:33 PM | JP | Jul 1, 2019 at 10:48:59 PM | JP |
| Perhaps try gluten free beer |  | 1 | 1 | Jul 2, 2019 at 2:39:15 PM | JP | Jul 2, 2019 at 2:39:28 PM | JP |
| Positive messages |  | 1 | 1 | Jul 2, 2019 at 12:33:51 AM | JP | Jul 2, 2019 at 12:34:04 AM | JP |
| Stretching exercise |  | 3 | 3 | Jul 5, 2019 at 2:33:38 PM | JP | Jul 5, 2019 at 9:49:49 PM | JP |
| Switch meds |  | 2 | 2 | Jul 2, 2019 at 2:08:36 PM | JP | Jul 5, 2019 at 1:56:18 PM | JP |
| Take control over your own treatment |  | 1 | 1 | Jul 2, 2019 at 4:56:11 PM | JP | Jul 2, 2019 at 4:56:26 PM | JP |
| Take OTC PRN for non-specific pain |  | 1 | 1 | Jul 1, 2019 at 10:47:40 PM | JP | Jul 1, 2019 at 10:47:59 PM | JP |
| Wristwarmer |  | 1 | 2 | Jul 5, 2019 at 2:28:57 PM | JP | Jul 5, 2019 at 2:32:44 PM | JP |
| Strategies for sleep hygeine |  | 3 | 3 | Jul 1, 2019 at 10:53:43 PM | JP | Jul 6, 2019 at 11:20:18 PM | JP |
| Pillow support |  | 1 | 1 | Jul 1, 2019 at 10:53:55 PM | JP | Jul 1, 2019 at 10:54:07 PM | JP |
| Strategies with intial diagnosis |  | 1 | 1 | Jul 1, 2019 at 11:51:03 PM | JP | Jul 5, 2019 at 9:14:44 PM | JP |
| Encouraging and positive messages |  | 4 | 20 | Jul 1, 2019 at 11:51:16 PM | JP | Jul 5, 2019 at 9:48:35 PM | JP |
| First year of diagnosis is the scariest part and you need time to get used to it |  | 2 | 2 | Jul 2, 2019 at 12:09:15 AM | JP | Jul 2, 2019 at 5:23:11 PM | JP |
| Meds will start help with time, less pain and function properly |  | 2 | 2 | Jul 2, 2019 at 12:09:15 AM | JP | Jul 7, 2019 at 12:28:56 AM | JP |
| You will learn how to work with 'bad days' |  | 1 | 1 | Jul 2, 2019 at 12:09:15 AM | JP | Jul 1, 2019 at 11:47:56 PM | JP |
| Get enough rest |  | 1 | 1 | Jul 2, 2019 at 4:52:54 PM | JP | Jul 2, 2019 at 4:53:04 PM | JP |
| Stress can make it worse |  | 1 | 1 | Jul 2, 2019 at 12:12:47 AM | JP | Jul 2, 2019 at 12:13:07 AM | JP |
| Worrying will make it worse |  | 1 | 1 | Jul 2, 2019 at 12:14:11 AM | JP | Jul 2, 2019 at 12:14:23 AM | JP |
| Trial and error |  | 1 | 1 | Jul 5, 2019 at 2:34:02 PM | JP | Jul 5, 2019 at 2:34:11 PM | JP |
| Try best to look after oneself |  | 1 | 1 | Jul 8, 2019 at 3:28:31 AM | JP | Jul 8, 2019 at 3:28:42 AM | JP |
| Try to stay active |  | 4 | 4 | Jul 8, 2019 at 3:35:46 AM | JP | Jul 8, 2019 at 3:29:04 AM | JP |
| Yoga |  | 1 | 3 | Jul 5, 2019 at 3:11:45 PM | JP | Jul 5, 2019 at 3:12:09 PM | JP |
| Strategies for doing things you love |  | 2 | 8 | Jul 5, 2019 at 9:50:18 PM | JP | Jul 6, 2019 at 11:56:20 PM | JP |
| Strategies for emotional distress |  | 3 | 5 | Jul 2, 2019 at 4:43:05 PM | JP | Jul 8, 2019 at 3:54:32 AM | JP |
| Accept some people won't understand |  | 2 | 2 | Jul 6, 2019 at 11:26:39 PM | JP | Jul 7, 2019 at 12:24:55 AM | JP |
| Accepted as what it is |  | 6 | 7 | Jul 1, 2019 at 10:26:35 PM | JP | Jul 8, 2019 at 3:54:13 AM | JP |
| Ask for specific things you need |  | 1 | 1 | Jul 7, 2019 at 12:08:35 AM | JP | Jul 7, 2019 at 12:08:50 AM | JP |
| Be careful with Internet |  | 2 | 12 | Jul 5, 2019 at 9:12:15 PM | JP | Jul 8, 2019 at 4:03:22 AM | JP |
| Counselling or therapy |  | 5 | 13 | Jul 3, 2019 at 9:43:49 AM | JP | Jul 7, 2019 at 12:31:33 AM | JP |
| Don't think about far future. Focus on today |  | 1 | 1 | Jul 3, 2019 at 10:16:37 AM | JP | Jul 3, 2019 at 10:16:55 AM | JP |
| Express to loved ones |  | 3 | 3 | Jul 5, 2019 at 9:23:48 PM | JP | Jul 7, 2019 at 12:02:17 AM | JP |
| Feel free to contact if you want to talk more |  | 4 | 5 | Jul 5, 2019 at 1:54:48 PM | JP | Jul 7, 2019 at 12:31:16 AM | JP |
| Gets better with time |  | 1 | 1 | Jul 3, 2019 at 10:14:13 AM | JP | Jul 3, 2019 at 10:14:23 AM | JP |
| Look after your mental health |  | 5 | 6 | Jul 2, 2019 at 4:50:24 PM | JP | Jul 8, 2019 at 3:25:40 AM | JP |
| mindfulness exercise |  | 3 | 4 | Jul 2, 2019 at 5:16:10 PM | JP | Jul 5, 2019 at 9:58:51 PM | JP |
| Offers resources |  | 6 | 9 | Jul 2, 2019 at 4:47:26 PM | JP | Jul 7, 2019 at 12:31:28 AM | JP |
| Provides positive messages or hope |  | 20 | 133 | Jul 1, 2019 at 10:45:59 PM | JP | Jul 8, 2019 at 4:05:10 AM | JP |
| Make most out of positive days |  | 7 | 10 | Jun 27, 2019 at 3:53:03 PM | JP | Jul 7, 2019 at 12:22:05 AM | JP |
| Set personal expectations |  | 2 | 3 | Jul 2, 2019 at 1:55:20 PM | JP | Jul 8, 2019 at 3:24:48 AM | JP |
| Put yourself first |  | 1 | 1 | Jul 2, 2019 at 5:07:06 PM | JP | Jul 2, 2019 at 5:07:15 PM | JP |
| Reach out to loved ones for support |  | 3 | 4 | Jul 3, 2019 at 9:46:33 AM | JP | Jul 7, 2019 at 12:02:26 AM | JP |
| Respect your limitations |  | 10 | 17 | Jul 5, 2019 at 2:19:33 PM | JP | Jul 8, 2019 at 3:27:36 AM | JP |
| Seek professional support |  | 2 | 2 | Jul 3, 2019 at 9:48:34 AM | JP | Jul 7, 2019 at 12:06:05 AM | JP |
| Self-care |  | 8 | 13 | Jul 5, 2019 at 8:48:29 PM | JP | Jul 8, 2019 at 3:27:44 AM | JP |
| Start meds |  | 1 | 2 | Jul 5, 2019 at 9:57:29 PM | JP | Jul 5, 2019 at 9:58:14 PM | JP |
| Support group |  | 3 | 3 | Jul 5, 2019 at 9:24:40 PM | JP | Jul 7, 2019 at 12:01:36 AM | JP |
| Try not to think about it |  | 2 | 3 | Jul 1, 2019 at 10:58:45 PM | JP | Jul 8, 2019 at 4:08:54 AM | JP |
| Try to stay active |  | 4 | 4 | Jul 8, 2019 at 3:35:34 AM | JP | Jul 8, 2019 at 3:29:04 AM | JP |
| Writing |  | 1 | 3 | Jul 5, 2019 at 8:48:07 PM | JP | Jul 5, 2019 at 8:57:05 PM | JP |
| Strategies for exercises |  | 1 | 1 | Jul 2, 2019 at 2:26:58 PM | JP | Jul 5, 2019 at 9:49:11 PM | JP |
| Be positive |  | 1 | 1 | Jul 2, 2019 at 2:27:51 PM | JP | Jul 2, 2019 at 2:29:32 PM | JP |
| Blood flow |  | 1 | 1 | Jul 2, 2019 at 2:30:41 PM | JP | Jul 2, 2019 at 2:30:51 PM | JP |
| Can exercise when using body correctly |  | 1 | 1 | Jul 2, 2019 at 2:30:54 PM | JP | Jul 2, 2019 at 2:31:04 PM | JP |
| Found another exercise that's satisfying |  | 4 | 15 | Jul 2, 2019 at 2:34:17 PM | JP | Jul 6, 2019 at 11:52:47 PM | JP |
| Hot bath |  | 1 | 1 | Jul 2, 2019 at 2:27:19 PM | JP | Jul 2, 2019 at 2:28:06 PM | JP |
| Sleep |  | 2 | 2 | Jul 2, 2019 at 2:27:36 PM | JP | Jul 5, 2019 at 9:38:18 PM | JP |
| Slowly go back to it |  | 1 | 3 | Jul 6, 2019 at 11:55:56 PM | JP | Jul 6, 2019 at 11:57:14 PM | JP |
| Tape up swollen joints |  | 1 | 1 | Jul 2, 2019 at 2:27:41 PM | JP | Jul 2, 2019 at 2:29:23 PM | JP |
| Strategies for family |  | 0 | 0 | Jun 27, 2019 at 3:59:54 PM | JP | Jul 8, 2019 at 3:50:42 AM | JP |
| Explain to family |  | 2 | 2 | Jul 2, 2019 at 5:06:15 PM | JP | Jul 5, 2019 at 9:29:13 PM | JP |
| If family doesn't understand |  | 1 | 2 | Jun 27, 2019 at 4:02:25 PM | JP | Jul 1, 2019 at 10:25:58 PM | JP |
| Guide them to different thread |  | 1 | 2 | Jul 5, 2019 at 3:43:06 PM | JP | Jul 5, 2019 at 3:43:41 PM | JP |
| Have a talk with them |  | 1 | 2 | Jul 5, 2019 at 3:45:54 PM | JP | Jul 5, 2019 at 3:46:13 PM | JP |
| If they don't understand you, bring them to your appointment |  | 2 | 3 | Jun 27, 2019 at 4:01:10 PM | JP | Jul 5, 2019 at 3:41:53 PM | JP |
| Limit contact with family |  | 2 | 2 | Jul 1, 2019 at 10:25:54 PM | JP | Jul 5, 2019 at 3:39:47 PM | JP |
| Set firm boundaries |  | 2 | 3 | Jul 1, 2019 at 10:27:22 PM | JP | Jul 5, 2019 at 3:40:24 PM | JP |
| Wishing that their author's family members can come and see how they do it in their family |  | 1 | 1 | Jun 27, 2019 at 4:05:28 PM | JP | Jun 27, 2019 at 4:05:28 PM | JP |
| Positive support of family really helps |  | 4 | 5 | Jul 1, 2019 at 10:27:59 PM | JP | Jul 8, 2019 at 3:50:38 AM | JP |
| Strategies for Partner |  | 0 | 0 | Jun 27, 2019 at 3:50:29 PM | JP | Jun 27, 2019 at 3:53:43 PM | JP |
| Have a conversation with partner |  | 1 | 1 | Jun 27, 2019 at 3:50:27 PM | JP | Jun 27, 2019 at 3:50:27 PM | JP |
| Support from family is helpful |  | 7 | 7 | Jul 2, 2019 at 5:08:30 PM | JP | Jul 7, 2019 at 12:11:36 AM | JP |
| Will burden family more if disease gets worse bc you didn't take care of yourself |  | 1 | 2 | Jul 3, 2019 at 9:48:51 AM | JP | Jul 3, 2019 at 10:15:10 AM | JP |
| Strategies for school |  | 1 | 1 | Jul 1, 2019 at 11:10:42 PM | JP | Jul 5, 2019 at 9:29:06 PM | JP |
| Had to unbrainwash myself that I'm not handicapped |  | 1 | 1 | Jul 1, 2019 at 11:10:49 PM | JP | Jul 1, 2019 at 11:11:14 PM | JP |
| Strategies for stress |  | 0 | 0 | Jul 1, 2019 at 10:47:16 PM | JP | Jul 1, 2019 at 10:47:20 PM | JP |
| Eliminate stressors |  | 1 | 1 | Jul 2, 2019 at 1:59:21 PM | JP | Jul 2, 2019 at 1:59:32 PM | JP |
| Try to keep stress levels moderated |  | 1 | 1 | Jul 1, 2019 at 10:47:26 PM | JP | Jul 1, 2019 at 10:49:02 PM | JP |
| Strategies for work |  | 2 | 4 | Jun 27, 2019 at 3:51:24 PM | JP | Jul 2, 2019 at 5:28:17 PM | JP |
| Exercise |  | 1 | 1 | Jul 1, 2019 at 10:50:56 PM | JP | Jul 1, 2019 at 10:51:17 PM | JP |
| Explain to work what's happening and request for accomodation |  | 5 | 7 | Jul 2, 2019 at 5:09:40 PM | JP | Jul 7, 2019 at 12:33:44 AM | JP |
| Go back to work when manageable |  | 1 | 1 | Jul 6, 2019 at 11:32:48 PM | JP | Jul 6, 2019 at 11:33:04 PM | JP |
| Moderated distraction helps at work |  | 1 | 1 | Jun 27, 2019 at 3:52:06 PM | JP | Jun 27, 2019 at 3:52:06 PM | JP |
| Reduce work |  | 1 | 1 | Jul 6, 2019 at 11:29:19 PM | JP | Jul 6, 2019 at 11:29:28 PM | JP |
| Shouldn't expect support from team at work |  | 1 | 4 | Jul 6, 2019 at 10:53:24 PM | JP | Jul 6, 2019 at 10:59:12 PM | JP |
| Strategies for working with or finding Health Care Professionals |  | 0 | 0 | Jul 1, 2019 at 10:44:49 PM | JP | Jul 1, 2019 at 10:45:01 PM | JP |
| Important to find HCP who listens and understands you |  | 2 | 3 | Jul 1, 2019 at 11:13:14 PM | JP | Jul 3, 2019 at 9:45:54 AM | JP |
| Incorporated orthopedist |  | 2 | 2 | Jul 1, 2019 at 10:45:07 PM | JP | Jul 5, 2019 at 9:34:50 PM | JP |
| Occupational therapist |  | 1 | 1 | Jul 5, 2019 at 2:29:50 PM | JP | Jul 5, 2019 at 2:30:06 PM | JP |
| Open Communication |  | 7 | 12 | Jul 2, 2019 at 12:04:23 AM | JP | Jul 8, 2019 at 4:06:55 AM | JP |
| Physiotherapist |  | 4 | 7 | Jul 5, 2019 at 2:32:29 PM | JP | Jul 6, 2019 at 11:57:09 PM | JP |
| Take control over your own treatment |  | 2 | 5 | Jul 2, 2019 at 1:57:46 PM | JP | Jul 2, 2019 at 4:56:08 PM | JP |
| Strategies to take meds |  | 0 | 0 | Jul 1, 2019 at 11:59:31 PM | JP | Jul 1, 2019 at 11:59:50 PM | JP |
| Anecdotes |  | 8 | 80 | Jul 3, 2019 at 4:22:18 PM | JP | Jul 7, 2019 at 12:28:27 AM | JP |
| Change diet |  | 1 | 1 | Jul 2, 2019 at 2:57:08 PM | JP | Jul 2, 2019 at 2:57:18 PM | JP |
| Education on meds (ex. side effects, tips) |  | 9 | 55 | Jul 2, 2019 at 4:40:34 PM | JP | Jul 7, 2019 at 12:24:38 AM | JP |
| If you don't take it, it'll get worse and burden loved ones more |  | 2 | 2 | Jul 3, 2019 at 9:49:23 AM | JP | Jul 8, 2019 at 4:07:46 AM | JP |
| Informed on risks of not taking meds |  | 1 | 2 | Jul 2, 2019 at 12:00:00 AM | JP | Jul 2, 2019 at 12:03:43 AM | JP |
| Meds are improving |  | 7 | 10 | Jul 2, 2019 at 12:23:11 AM | JP | Jul 8, 2019 at 4:05:25 AM | JP |
| Meds will kick in with time |  | 6 | 7 | Jul 2, 2019 at 1:39:24 PM | JP | Jul 8, 2019 at 4:01:57 AM | JP |
| Open communication with HCP |  | 9 | 21 | Jul 2, 2019 at 12:04:03 AM | JP | Jul 6, 2019 at 11:27:36 PM | JP |
| Switch or add or modify meds |  | 9 | 22 | Jul 2, 2019 at 12:06:17 AM | JP | Jul 5, 2019 at 8:58:09 PM | JP |
| Understand that finding the right treatment may take time |  | 10 | 22 | Jul 2, 2019 at 4:40:08 PM | JP | Jul 8, 2019 at 3:54:07 AM | JP |
| Struggles taking medications |  | 0 | 0 | Jul 1, 2019 at 11:58:24 PM | JP | Jul 2, 2019 at 2:56:47 PM | JP |
| Because of someone they know |  | 1 | 1 | Jul 3, 2019 at 4:14:11 PM | JP | Jul 3, 2019 at 4:14:39 PM | JP |
| Because loved one with the same disease died from the med |  | 1 | 1 | Jul 1, 2019 at 11:58:56 PM | JP | Jul 1, 2019 at 11:59:23 PM | JP |
| Cost concern |  | 1 | 2 | Jul 3, 2019 at 9:41:27 AM | JP | Jul 3, 2019 at 9:44:02 AM | JP |
| Hard time taking the pills |  | 1 | 1 | Jul 2, 2019 at 2:06:50 PM | JP | Jul 6, 2019 at 11:44:04 PM | JP |
| Limited options due to med condition |  | 1 | 1 | Jul 2, 2019 at 2:58:08 PM | JP | Jul 2, 2019 at 2:58:21 PM | JP |
| Medications |  | 0 | 0 | Jul 1, 2019 at 10:37:54 PM | JP | Jul 1, 2019 at 10:38:03 PM | JP |
| Negative experiences with meds (Side effects or Allergies or intolerance) |  | 9 | 37 | Jul 1, 2019 at 10:38:11 PM | JP | Jul 7, 2019 at 12:10:39 AM | JP |
| Not a fan of meds |  | 1 | 1 | Jul 1, 2019 at 10:36:38 PM | JP | Jul 1, 2019 at 10:36:49 PM | JP |
| Medications stopped working |  | 5 | 8 | Jul 2, 2019 at 2:07:46 PM | JP | Jul 6, 2019 at 11:54:25 PM | JP |
| Overwhelmed with amount of meds |  | 3 | 4 | Jul 2, 2019 at 2:06:38 PM | JP | Jul 6, 2019 at 11:44:04 PM | JP |
| Running out of options |  | 2 | 2 | Jul 2, 2019 at 2:17:49 PM | JP | Jul 6, 2019 at 11:53:33 PM | JP |
| Scared to take meds |  | 1 | 7 | Jul 3, 2019 at 4:10:56 PM | JP | Jul 3, 2019 at 4:21:59 PM | JP |
| The extent and effect of modern medicine |  | 1 | 1 | Jul 2, 2019 at 12:21:49 AM | JP | Jul 2, 2019 at 12:22:21 AM | JP |
| Struggles with disease |  | 0 | 0 | Jul 1, 2019 at 11:21:01 PM | JP | Jul 8, 2019 at 3:20:21 AM | JP |
| Can't drink alcohol |  | 1 | 3 | Jul 2, 2019 at 2:37:03 PM | JP | Jul 2, 2019 at 2:38:20 PM | JP |
| Fatigue |  | 5 | 12 | Jul 2, 2019 at 12:29:17 AM | JP | Jul 6, 2019 at 11:58:43 PM | JP |
| Feeling useless and can't do anything about it |  | 2 | 2 | Jul 2, 2019 at 1:44:06 PM | JP | Jul 7, 2019 at 12:04:46 AM | JP |
| Strategies tried before but wasn't successful |  | 1 | 1 | Jul 2, 2019 at 1:44:59 PM | JP | Jul 2, 2019 at 1:45:29 PM | JP |
| Functional limitation |  | 4 | 10 | Jul 2, 2019 at 5:16:44 PM | JP | Jul 8, 2019 at 4:06:03 AM | JP |
| Hard to find help |  | 1 | 1 | Jul 8, 2019 at 3:20:21 AM | JP | Jul 8, 2019 at 3:20:06 AM | JP |
| Hard to find resources relevant to them |  | 2 | 2 | Jul 8, 2019 at 3:19:24 AM | JP | Jul 3, 2019 at 9:37:49 AM | JP |
| Hard to find time to manage disease |  | 1 | 3 | Jul 2, 2019 at 5:12:08 PM | JP | Jul 2, 2019 at 5:12:48 PM | JP |
| Hard to maintain diet |  | 1 | 3 | Jul 5, 2019 at 9:38:56 PM | JP | Jul 5, 2019 at 9:41:37 PM | JP |
| Inflammation |  | 4 | 5 | Jul 2, 2019 at 2:33:32 PM | JP | Jul 5, 2019 at 8:40:25 PM | JP |
| Pain |  | 4 | 6 | Jul 1, 2019 at 11:21:08 PM | JP | Jul 5, 2019 at 9:44:09 PM | JP |
| In so much pain |  | 2 | 2 | Jul 1, 2019 at 10:35:33 PM | JP | Jul 2, 2019 at 12:18:54 AM | JP |
| Non-adherent |  | 2 | 2 | Jul 2, 2019 at 2:10:55 PM | JP | Jul 3, 2019 at 9:31:15 AM | JP |
| Weight Gain |  | 1 | 1 | Jul 5, 2019 at 3:07:57 PM | JP | Jul 5, 2019 at 3:08:13 PM | JP |
| Struggles with doing activities you love |  | 2 | 2 | Jul 2, 2019 at 2:08:59 PM | JP | Jul 5, 2019 at 3:08:22 PM | JP |
| Sports |  | 0 | 0 | Jul 2, 2019 at 2:09:27 PM | JP | Jul 2, 2019 at 2:09:30 PM | JP |
| Can't do things I love |  | 4 | 9 | Jul 2, 2019 at 2:13:24 PM | JP | Jul 7, 2019 at 12:04:57 AM | JP |
| Continued to play |  | 1 | 1 | Jul 2, 2019 at 2:09:36 PM | JP | Jul 6, 2019 at 11:44:04 PM | JP |
| Feeling depressed |  | 2 | 4 | Jul 2, 2019 at 2:14:55 PM | JP | Jul 6, 2019 at 11:46:02 PM | JP |
| Felt like letting team down |  | 1 | 1 | Jul 2, 2019 at 2:09:54 PM | JP | Jul 2, 2019 at 2:10:19 PM | JP |
| People don't understanding |  | 1 | 1 | Jul 2, 2019 at 2:12:06 PM | JP | Jul 2, 2019 at 2:12:29 PM | JP |
| Tried other exercises |  | 1 | 3 | Jul 2, 2019 at 2:35:31 PM | JP | Jul 2, 2019 at 2:38:02 PM | JP |
| Struggles with exercising |  | 0 | 0 | Jul 1, 2019 at 11:04:36 PM | JP | Jul 1, 2019 at 11:04:47 PM | JP |
| Can't meet expectations of healthy individuals or peers or friends |  | 1 | 1 | Jul 1, 2019 at 11:06:03 PM | JP | Jul 1, 2019 at 11:06:36 PM | JP |
| Depressed because could not exercise |  | 3 | 4 | Jul 1, 2019 at 11:05:17 PM | JP | Jul 5, 2019 at 9:41:58 PM | JP |
| Very weak because lack of exercise |  | 2 | 4 | Jul 1, 2019 at 11:11:29 PM | JP | Jul 6, 2019 at 11:57:44 PM | JP |
| Struggles with family |  | 3 | 3 | Jun 27, 2019 at 3:57:13 PM | JP | Jul 5, 2019 at 9:57:55 PM | JP |
| Can I expand my family |  | 1 | 3 | Jul 5, 2019 at 10:05:51 PM | JP | Jul 5, 2019 at 10:07:02 PM | JP |
| Family doesn't understand |  | 3 | 5 | Jul 3, 2019 at 4:12:54 PM | JP | Jul 8, 2019 at 3:49:38 AM | JP |
| Don't understand what's happening |  | 2 | 2 | Jul 2, 2019 at 12:32:02 AM | JP | Jul 3, 2019 at 4:13:26 PM | JP |
| Father doesn't understand why you can't push through the day |  | 1 | 1 | Jun 27, 2019 at 3:57:37 PM | JP | Jun 27, 2019 at 3:57:37 PM | JP |
| Mother doesn't understand |  | 1 | 2 | Jun 27, 2019 at 4:08:45 PM | JP | Jul 8, 2019 at 3:47:22 AM | JP |
| Family is pushing to do regular activities |  | 1 | 1 | Jul 2, 2019 at 12:31:15 AM | JP | Jul 2, 2019 at 12:31:31 AM | JP |
| Family member who also has RA but doesn't understand |  | 1 | 4 | Jul 5, 2019 at 3:15:43 PM | JP | Jul 5, 2019 at 3:18:38 PM | JP |
| Feels guilty to put burden on family |  | 4 | 6 | Jul 3, 2019 at 9:36:41 AM | JP | Jul 8, 2019 at 4:08:08 AM | JP |
| Heartbreaking to not being fully committed to family |  | 1 | 6 | Jul 2, 2019 at 5:03:43 PM | JP | Jul 5, 2019 at 3:45:40 PM | JP |
| Feels guilty because family is picking up slack |  | 1 | 1 | Jun 27, 2019 at 4:04:55 PM | JP | Jun 27, 2019 at 4:04:55 PM | JP |
| Struggles with Health Care Professionals |  | 0 | 0 | Jul 1, 2019 at 10:30:50 PM | JP | Jul 1, 2019 at 10:31:03 PM | JP |
| asking for help |  | 1 | 2 | Jul 5, 2019 at 9:39:47 PM | JP | Jul 5, 2019 at 9:46:56 PM | JP |
| Developed phobia of needles |  | 1 | 1 | Jul 3, 2019 at 9:35:47 AM | JP | Jul 3, 2019 at 9:36:04 AM | JP |
| Didn't help |  | 1 | 1 | Jul 3, 2019 at 9:45:24 AM | JP | Jul 3, 2019 at 9:45:34 AM | JP |
| Dr is not listening to me OR not understanding me |  | 3 | 5 | Jul 1, 2019 at 10:31:09 PM | JP | Jul 6, 2019 at 11:59:44 PM | JP |
| Frustrated when Dr doesn't know what to do |  | 2 | 3 | Jul 1, 2019 at 11:00:45 PM | JP | Jul 2, 2019 at 2:50:27 PM | JP |
| Hard to find new Dr who can understand |  | 2 | 4 | Jul 1, 2019 at 10:32:18 PM | JP | Jul 3, 2019 at 9:50:37 AM | JP |
| Long wait times |  | 1 | 2 | Jul 2, 2019 at 12:16:15 AM | JP | Jul 2, 2019 at 12:16:48 AM | JP |
| Wasn't diagnosed correctly and was left untreated |  | 1 | 1 | Jul 1, 2019 at 11:19:27 PM | JP | Jul 1, 2019 at 11:20:09 PM | JP |
| When to contact HCP |  | 1 | 1 | Jul 5, 2019 at 3:12:48 PM | JP | Jul 5, 2019 at 3:13:00 PM | JP |
| Struggles with initial diagnosis |  | 0 | 0 | Jul 1, 2019 at 11:46:24 PM | JP | Jul 1, 2019 at 11:47:14 PM | JP |
| Alot of things change |  | 1 | 1 | Jul 2, 2019 at 12:10:20 AM | JP | Jul 2, 2019 at 12:10:30 AM | JP |
| Didn't know what to expect |  | 2 | 3 | Jul 2, 2019 at 2:05:47 PM | JP | Jul 6, 2019 at 11:44:04 PM | JP |
| Suicidal Thoughts |  | 1 | 5 | Jul 1, 2019 at 11:46:40 PM | JP | Jul 1, 2019 at 11:31:57 PM | JP |
| Don't want people close to me to suffer if I die |  | 1 | 1 | Jul 1, 2019 at 11:46:40 PM | JP | Jul 1, 2019 at 11:22:44 PM | JP |
| Seeks for thoughts or opinions |  | 1 | 1 | Jul 1, 2019 at 11:46:40 PM | JP | Jul 1, 2019 at 11:23:30 PM | JP |
| Thinking about shortened life expectancy with the disease |  | 1 | 1 | Jul 1, 2019 at 11:46:40 PM | JP | Jul 1, 2019 at 11:22:13 PM | JP |
| Why me |  | 3 | 3 | Jul 2, 2019 at 12:09:44 AM | JP | Jul 5, 2019 at 9:10:50 PM | JP |
| Struggles with sleep |  | 1 | 3 | Jul 1, 2019 at 11:03:20 PM | JP | Jul 1, 2019 at 11:04:23 PM | JP |
| Struggles with work |  | 7 | 14 | Jul 1, 2019 at 11:32:50 PM | JP | Jul 7, 2019 at 12:10:01 AM | JP |
| Anxiety |  | 1 | 1 | Jul 1, 2019 at 11:33:11 PM | JP | Jul 1, 2019 at 11:33:26 PM | JP |
| Coworkers don't understand or not supportive |  | 2 | 6 | Jul 6, 2019 at 11:01:15 PM | JP | Jul 6, 2019 at 11:25:08 PM | JP |
| Exhausted |  | 1 | 2 | Jul 6, 2019 at 11:09:34 PM | JP | Jul 6, 2019 at 11:32:18 PM | JP |
| Requires alot of energy |  | 2 | 5 | Jul 2, 2019 at 5:02:22 PM | JP | Jul 6, 2019 at 11:31:49 PM | JP |
| Work cannot accomodate |  | 1 | 2 | Jul 6, 2019 at 11:29:41 PM | JP | Jul 6, 2019 at 11:30:47 PM | JP |
| Struggles-Emotional toll |  | 0 | 0 | Jun 27, 2019 at 3:54:17 PM | JP | Jul 8, 2019 at 3:54:42 AM | JP |
| Acceptance is hard |  | 4 | 10 | Jul 5, 2019 at 9:26:50 PM | JP | Jul 8, 2019 at 3:53:20 AM | JP |
| Anxious |  | 4 | 5 | Jul 2, 2019 at 4:41:41 PM | JP | Jul 5, 2019 at 8:58:58 PM | JP |
| Being called lazy, or getting acused for not trying hard |  | 5 | 7 | Jul 2, 2019 at 1:59:54 PM | JP | Jul 8, 2019 at 3:49:19 AM | JP |
| Big toll on mental health |  | 1 | 1 | Jun 26, 2019 at 11:43:06 AM | JP | Jun 26, 2019 at 11:43:06 AM | JP |
| Cried |  | 2 | 3 | Jul 5, 2019 at 9:18:31 PM | JP | Jul 6, 2019 at 11:10:31 PM | JP |
| defeated |  | 1 | 1 | Jul 5, 2019 at 8:51:12 PM | JP | Jul 6, 2019 at 11:36:02 PM | JP |
| Depressed |  | 6 | 9 | Jul 3, 2019 at 9:31:51 AM | JP | Jul 6, 2019 at 11:29:01 PM | JP |
| Discouraged |  | 2 | 2 | Jul 5, 2019 at 2:14:06 PM | JP | Jul 6, 2019 at 11:19:00 PM | JP |
| Don't believe disease will get better |  | 2 | 2 | Jul 2, 2019 at 5:22:23 PM | JP | Jul 5, 2019 at 10:10:55 PM | JP |
| Don't know what to do |  | 1 | 2 | Jul 1, 2019 at 10:35:26 PM | JP | Jul 1, 2019 at 10:39:48 PM | JP |
| Don't want people to feel sorry |  | 1 | 1 | Jul 2, 2019 at 2:15:45 PM | JP | Jul 2, 2019 at 2:16:02 PM | JP |
| Don't want to show to loved ones |  | 1 | 1 | Jul 2, 2019 at 4:36:32 PM | JP | Jul 2, 2019 at 4:36:59 PM | JP |
| Feels alone |  | 3 | 3 | Jul 3, 2019 at 4:20:24 PM | JP | Jul 6, 2019 at 11:26:59 PM | JP |
| Feels hopeless and lost |  | 7 | 12 | Jul 2, 2019 at 12:18:14 AM | JP | Jul 6, 2019 at 11:36:07 PM | JP |
| Feels invisible |  | 1 | 1 | Jul 6, 2019 at 11:03:36 PM | JP | Jul 6, 2019 at 11:04:07 PM | JP |
| Frustrated |  | 4 | 7 | Jul 5, 2019 at 2:12:21 PM | JP | Jul 6, 2019 at 11:06:49 PM | JP |
| Gets worried |  | 2 | 4 | Jul 2, 2019 at 12:17:46 AM | JP | Jul 6, 2019 at 11:19:04 PM | JP |
| Hard to ask for support |  | 1 | 2 | Jul 6, 2019 at 11:04:50 PM | JP | Jul 6, 2019 at 11:05:25 PM | JP |
| Hard to find resources relevant to them |  | 2 | 2 | Jul 8, 2019 at 3:19:28 AM | JP | Jul 3, 2019 at 9:37:49 AM | JP |
| Hard to not let RA define me |  | 1 | 1 | Jul 5, 2019 at 10:11:12 PM | JP | Jul 5, 2019 at 10:11:27 PM | JP |
| Having terrible time |  | 1 | 1 | Jul 1, 2019 at 10:35:13 PM | JP | Jul 1, 2019 at 10:35:59 PM | JP |
| Internet search shows scary results |  | 1 | 2 | Jul 5, 2019 at 9:09:57 PM | JP | Jul 5, 2019 at 9:13:46 PM | JP |
| Lack of fulfillment |  | 1 | 1 | Jul 2, 2019 at 1:50:32 PM | JP | Jul 8, 2019 at 3:25:00 AM | JP |
| Major adjustment in life |  | 1 | 1 | Jul 6, 2019 at 11:30:16 PM | JP | Jul 6, 2019 at 11:30:29 PM | JP |
| Missing out on life |  | 2 | 2 | Jul 2, 2019 at 5:29:10 PM | JP | Jul 5, 2019 at 10:13:57 PM | JP |
| Need better motivation |  | 1 | 1 | Jul 3, 2019 at 9:30:47 AM | JP | Jul 3, 2019 at 9:30:59 AM | JP |
| No Inperson support group available |  | 1 | 1 | Jul 8, 2019 at 3:21:16 AM | JP | Jul 8, 2019 at 3:21:11 AM | JP |
| Noone who can relate to them |  | 1 | 1 | Jul 3, 2019 at 9:37:53 AM | JP | Jul 3, 2019 at 9:38:14 AM | JP |
| Overestimating ability |  | 1 | 1 | Jul 5, 2019 at 10:01:24 PM | JP | Jul 5, 2019 at 10:04:24 PM | JP |
| People don't think chronic illness is serious |  | 3 | 7 | Jul 6, 2019 at 10:59:47 PM | JP | Jul 8, 2019 at 3:49:51 AM | JP |
| Rant or Vent |  | 7 | 9 | Jul 2, 2019 at 2:51:26 PM | JP | Jul 6, 2019 at 11:43:33 PM | JP |
| Sad |  | 3 | 3 | Jul 5, 2019 at 9:00:00 PM | JP | Jul 5, 2019 at 9:55:33 PM | JP |
| scared |  | 5 | 8 | Jul 2, 2019 at 4:35:24 PM | JP | Jul 7, 2019 at 12:13:29 AM | JP |
| Scared about future |  | 3 | 3 | Jul 1, 2019 at 10:39:56 PM | JP | Jul 3, 2019 at 9:32:08 AM | JP |
| Stressed |  | 2 | 2 | Jul 2, 2019 at 1:52:07 PM | JP | Jul 3, 2019 at 9:31:23 AM | JP |
| Struggling |  | 3 | 3 | Jun 26, 2019 at 11:42:36 AM | JP | Jul 6, 2019 at 11:03:30 PM | JP |
| Sucks |  | 1 | 2 | Jul 2, 2019 at 5:00:03 PM | JP | Jul 2, 2019 at 5:00:20 PM | JP |
| Suicidal thoughts |  | 2 | 2 | Jul 5, 2019 at 3:20:44 PM | JP | Jul 7, 2019 at 12:00:04 AM | JP |
| Taking antidepressants |  | 1 | 1 | Jul 2, 2019 at 1:40:02 PM | JP | Jul 2, 2019 at 1:40:30 PM | JP |
| tired |  | 5 | 6 | Jul 2, 2019 at 2:47:52 PM | JP | Jul 6, 2019 at 11:09:12 PM | JP |
| Tough |  | 1 | 1 | Jun 27, 2019 at 3:51:18 PM | JP | Jun 27, 2019 at 3:51:18 PM | JP |
| Tried everything but is not working |  | 1 | 1 | Jul 2, 2019 at 2:17:28 PM | JP | Jul 2, 2019 at 2:17:42 PM | JP |
| What should my realistic expectation be |  | 1 | 1 | Jul 5, 2019 at 2:12:50 PM | JP | Jul 5, 2019 at 2:13:04 PM | JP |
| Why me |  | 2 | 2 | Jul 3, 2019 at 4:20:45 PM | JP | Jul 5, 2019 at 9:10:36 PM | JP |
| worn out |  | 3 | 3 | Jul 2, 2019 at 12:27:50 AM | JP | Jul 5, 2019 at 3:21:26 PM | JP |
| Struggling in school |  | 2 | 2 | Jun 26, 2019 at 11:44:27 AM | JP | Jul 3, 2019 at 9:34:15 AM | JP |
| Difficult emotionally back in chilldhood andor teen years |  | 1 | 1 | Jul 1, 2019 at 11:08:04 PM | JP | Jul 1, 2019 at 11:08:29 PM | JP |
| No energy to fully participate in school activities |  | 1 | 2 | Jul 2, 2019 at 12:30:24 AM | JP | Jul 2, 2019 at 1:46:29 PM | JP |
| Was bullied for limping |  | 1 | 1 | Jul 1, 2019 at 11:08:44 PM | JP | Jul 1, 2019 at 11:09:15 PM | JP |
| Suicidal Thoughts |  | 2 | 7 | Jul 1, 2019 at 11:19:04 PM | JP | Jul 3, 2019 at 9:37:13 AM | JP |
| Commenters admiting they also had suicidal thoughts |  | 2 | 10 | Jul 2, 2019 at 12:06:44 AM | JP | Jul 3, 2019 at 9:46:24 AM | JP |
| Don't want people close to me to suffer if I die |  | 1 | 1 | Jul 1, 2019 at 11:22:29 PM | JP | Jul 1, 2019 at 11:22:44 PM | JP |
| Don't want to live if completely physically disabled |  | 2 | 3 | Jul 1, 2019 at 11:55:20 PM | JP | Jul 7, 2019 at 12:00:34 AM | JP |
| Lost a friend due to disease |  | 1 | 1 | Jul 2, 2019 at 12:24:39 AM | JP | Jul 2, 2019 at 12:25:11 AM | JP |
| Seeks for thoughts or opinions |  | 1 | 1 | Jul 1, 2019 at 11:23:14 PM | JP | Jul 1, 2019 at 11:23:30 PM | JP |
| Thinking about shortened life expectancy with the disease |  | 1 | 1 | Jul 1, 2019 at 11:21:43 PM | JP | Jul 1, 2019 at 11:22:13 PM | JP |
| We don't know how much meds will improve or new treatments will arise |  | 1 | 3 | Jul 2, 2019 at 12:00:29 AM | JP | Jul 2, 2019 at 12:27:28 AM | JP |
| Thanks |  | 0 | 0 | Jul 1, 2019 at 10:55:41 PM | JP | Jul 8, 2019 at 3:16:10 AM | JP |
| Appreciates support from government |  | 1 | 1 | Jul 2, 2019 at 1:53:02 PM | JP | Jul 2, 2019 at 1:53:33 PM | JP |
| Appreciates support of health care |  | 2 | 2 | Jul 2, 2019 at 1:53:12 PM | JP | Jul 7, 2019 at 12:11:54 AM | JP |
| Thanks community for support |  | 17 | 44 | Jul 1, 2019 at 10:26:57 PM | JP | Jul 7, 2019 at 12:32:53 AM | JP |
| Thanks sharing tips for disease management |  | 10 | 20 | Jul 1, 2019 at 10:55:26 PM | JP | Jul 7, 2019 at 12:28:08 AM | JP |
| Tips on exercising |  | 1 | 1 | Jul 1, 2019 at 11:10:05 PM | JP | Jul 1, 2019 at 11:10:21 PM | JP |
| Use of CBD |  | 1 | 1 | Jul 2, 2019 at 2:53:15 PM | JP | Jul 2, 2019 at 2:53:47 PM | JP |
| Effectiveness |  | 1 | 2 | Jul 2, 2019 at 2:54:10 PM | JP | Jul 2, 2019 at 2:54:36 PM | JP |
| What 'bad day' feels like |  | 1 | 1 | Jul 1, 2019 at 10:53:18 PM | JP | Jul 1, 2019 at 10:53:34 PM | JP |
